# The BAARA (Biological AutomAted RAdiotracking) System: A New Approach in Ecological Field Studies

**DOI:** 10.1371/journal.pone.0116785

**Published:** 2015-02-25

**Authors:** Šimon Řeřucha, Tomáš Bartonička, Petr Jedlička, Martin Čížek, Ondřej Hlouša, Radek Lučan, Ivan Horáček

**Affiliations:** 1 Institute of Scientific Instruments of the ASCR, v.v.i., Královopolská 147, CZ 612 64 Brno, Czech Republic; 2 Department of Botany and Zoology, Masaryk University, Kotlářská 2, CZ 611 37 Brno, Czech Republic; 3 Department of Zoology, Charles University in Prague, Viničná 7, CZ 12844 Praha, Czech Republic; Pacific Northwest National Laboratory, UNITED STATES

## Abstract

Radiotracking is an important and often the only possible method to explore specific habits and the behaviour of animals, but it has proven to be very demanding and time-consuming, especially when frequent positioning of a large group is required. Our aim was to address this issue by making the process partially automated, to mitigate the demands and related costs. This paper presents a novel automated tracking system that consists of a network of automated tracking stations deployed within the target area. Each station reads the signals from telemetry transmitters, estimates the bearing and distance of the tagged animals and records their position. The station is capable of tracking a theoretically unlimited number of transmitters on different frequency channels with the period of 5–15 seconds per single channel. An ordinary transmitter that fits within the supported frequency band might be used with BAARA (Biological AutomAted RAdiotracking); an extra option is the use of a custom-programmable transmitter with configurable operational parameters, such as the precise frequency channel or the transmission parameters. This new approach to a tracking system was tested for its applicability in a series of field and laboratory tests. BAARA has been tested within fieldwork explorations of *Rousettus aegyptiacus* during field trips to Dakhla oasis in Egypt. The results illustrate the novel perspective which automated radiotracking opens for the study of spatial behaviour, particularly in addressing topics in the domain of population ecology.

## Introduction

Since many wildlife species are secretive and difficult to observe, radio tracking is a valuable tool to learn more about their respective life histories. As a result, radio tracking studies are very common throughout the current wildlife literature e.g. [1–3].

During the last 50 years, biologists have attached radio transmitters to animals to monitor their locations and movements [4]. Positioning was initially performed manually, but the requirement for automation soon arose. In the 1970s, it became possible to track large animals by satellite technology (NIMBUS and ARGOS systems); more recently, the GNSS (Global Navigation Satellite System) systems expanded the available options. Unfortunately, due to the size constraints of satellite transmitters/receivers, VHF (Very High Frequency) radio tracking remains the sole means to the track small animals [5–7].

A promising alternative is the use of automated telemetry systems. Such tracking systems and bearing estimation started in the 1960s [8]. Further radiotracking systems have been introduced by [9–12]. Recently, another automated radio tracking system (ARTS) that monitors the behaviour of wild animals in rainforest environments has been developed [7]. The major disadvantage of all these systems is their static deployment in a particular limited area and their installation in a different area is very demanding.

Our initial motivation was the observation of a demanding model group, represented by the Egyptian fruit bat (*Rousettus aegyptiacus*), in several locations. The body mass of fruit bats limits the use of satellite tracking, since the transmitters are generally too heavy or have a limited lifetime. Their nocturnal activity restricts the use of solar recharging and their high mobility [13] requires rather frequent positioning and large terrain coverage. The next important aspect was the remote habitats where we carried out the studies, which required an easy transportability of the tracking equipment. Finally, funding issues limited the available man-days.

To address these constraints, we have designed a new telemetry system, referred to as BAARA (Biological AutomAted RAdiotracking). The principal components of BARRA are light-weight tracking stations (less than 5 kg each), which are easily transported and deployed in the area of interest. The stations read the signals from telemetry transmitters, typically over a distance of several km (depending on the transmitter) and record their position. These primary position data from multiple stations and manual tracking are later combined to obtain more precise positional information.

Biological automated radiotracking is primarily intended as a supplement to manual tracking: the automatic system continuously tracks the entire set of monitored individuals, whereas manual tracking is used to collect additional information such as feeding and roosting sites, as well as terrain specifics. In this article, we present the key concepts, principles and performance assessment of the BAARA system. First, we provide an overview of the system architecture and especially the localisation techniques and related data processing. Then we present the testing and calibration measurements we have carried out to assess the system feasibility and performance. Finally, the discussion deals with both theoretical practical limitations posed by the chosen methods and its advantages, since, we believe that the concept represents a major step towards improvements in methodology for radiotracking in zoological studies.

## Material and Methods

### Ethics Statement

All manipulations with *R. aegyptiacus* were conducted under the permission granted by the Nature Conservation Egypt (No. 22408921, 546223). The authors have been authorized to manipulate with free-living bats according to the certificate of competency No 104/2002-V4 (§ 17 of the law No 246/1992). The field studies have been carried out in the Dakhla Oasis (25°41′N, 28°52′E), where 111 individual animals were tagged by BAARA system and by homing in method. A 7 g processor-radio-transmitter Biotag (Institute of Scientific Instruments, Academy of Sciences of the Czech Republic) was glued using surgical adhesive (Universum, Universum Ltd., Czech Republic) to the interscapular region of each bat after trimming the fur. The transmitter always amounted to less than 5% of a bat’s body mass.

No specific permissions were required for field deployment of BAARA in southern Turkey, near Adana (Sayköy, 36°56′N, 34°47′E), because transmitter was not attached on bats. This field study did not involve endangered or protected species.

### Transmitter localisation

**Fig 1:**
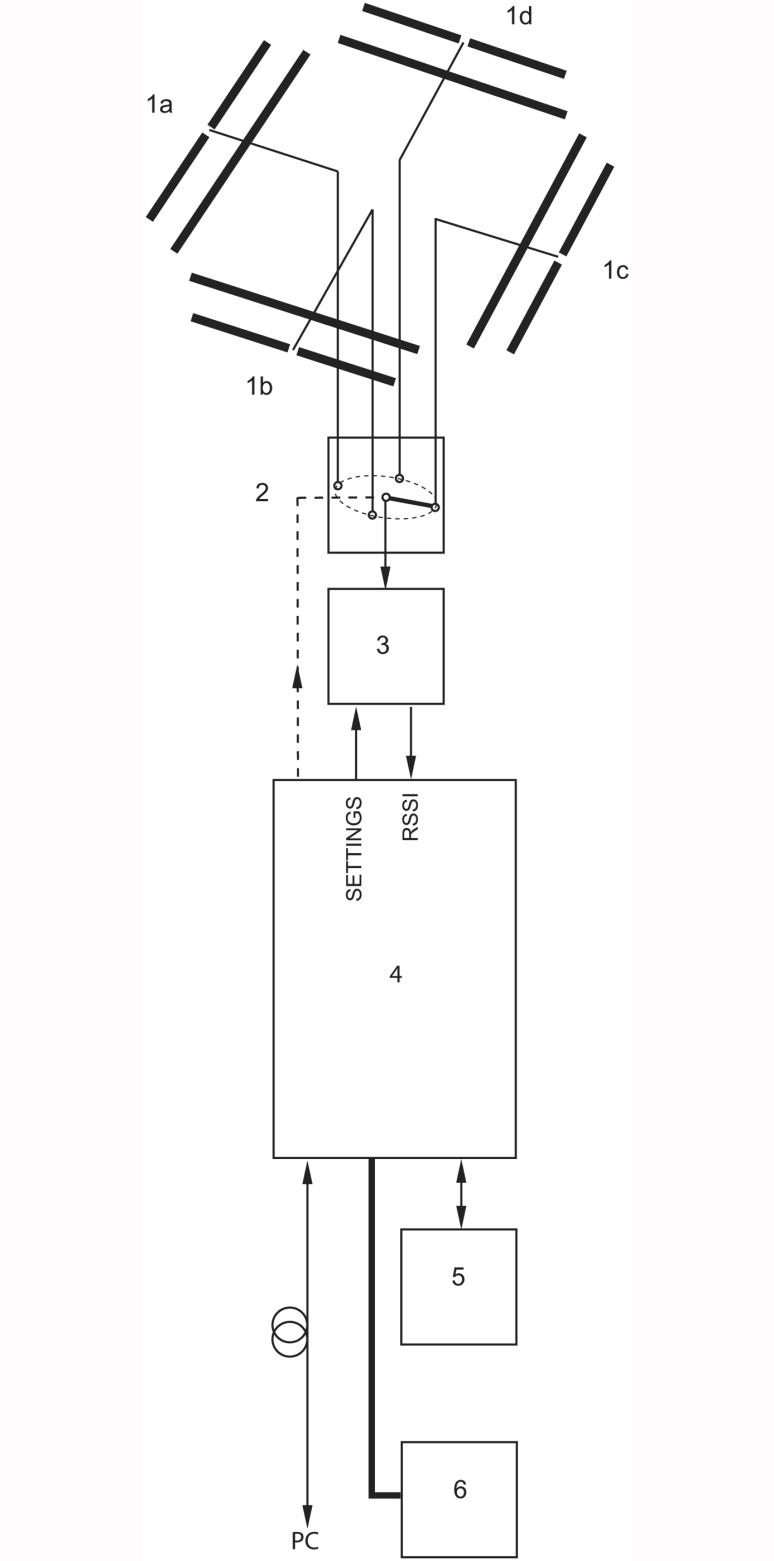
Block diagram of the BAARA system. 1-omnidirectional antenna array consisting of four directional antennas, 2-antenna multiplexing switch, 3-digitally controlled one channel receiver, 4-main control unit (microcontroller based) driving the antenna system and receiver as well as processing the incoming telemetry signals, 5-memory (SD-card) for data recording, 6-lithium-polymer accumulator for field operation.

**Fig 2:**
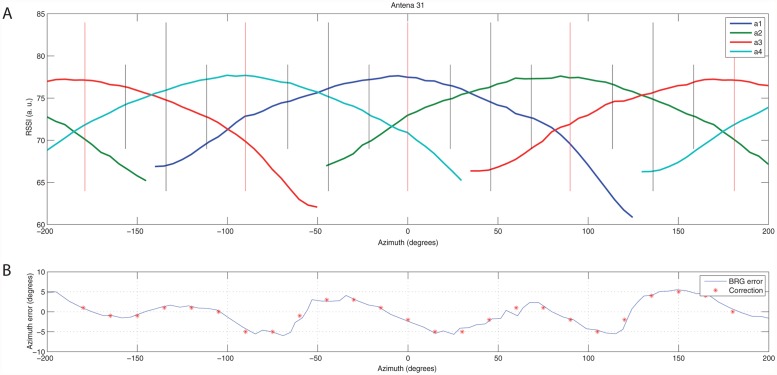
Observed radiation pattern. a) Observed radiation pattern of a particular antenna array. Sinusoidal patterns allow the detection algorithm to identify the azimuth of incoming signal unambiguously, b) Azimuth calculation error (denoted as BRG error) caused by the antenna pattern imperfections. An inverse function then forms a calibration vector that is applied to all signal readings in order to cancel out the influence of the imperfections.

The localisation is based on a time-multiplexed reception of the radio signal (from the telemetry transmitters) with an antenna array that is organised in a way that ensures 360 degree coverage. The array consists of four directional antennas (Fig. 1), where the axes of the front lobes are mutually perpendicular, i.e., one antenna is oriented northwards, one westwards, one eastwards and one southwards. The radiation pattern is adjusted so that its front half is approximately sinusoidal, as shown in Fig. 2a, where the gain of particular antennas depending on the incident angle of radio signal is shown. When the radiation pattern is not optimal, e.g., due to interactions among the individual antennas, the imprecision is compensated for later within the evaluation.

The RF (radio frequency) signal from antennas is read by a single-channel radio receiver that measures the received signal strength (RSSI). The antenna inputs are multiplexed in a round-robin manner (typically ~15 ms per cycle or ~4 ms per antenna), so that a simultaneous reception is simulated. The acquired RSSI recording (typically spanning 3–10 transmitter “beeps”, i.e., 5–20 s) is then used for the estimation of the transmitter position relative to the station position.

**Fig 3:**
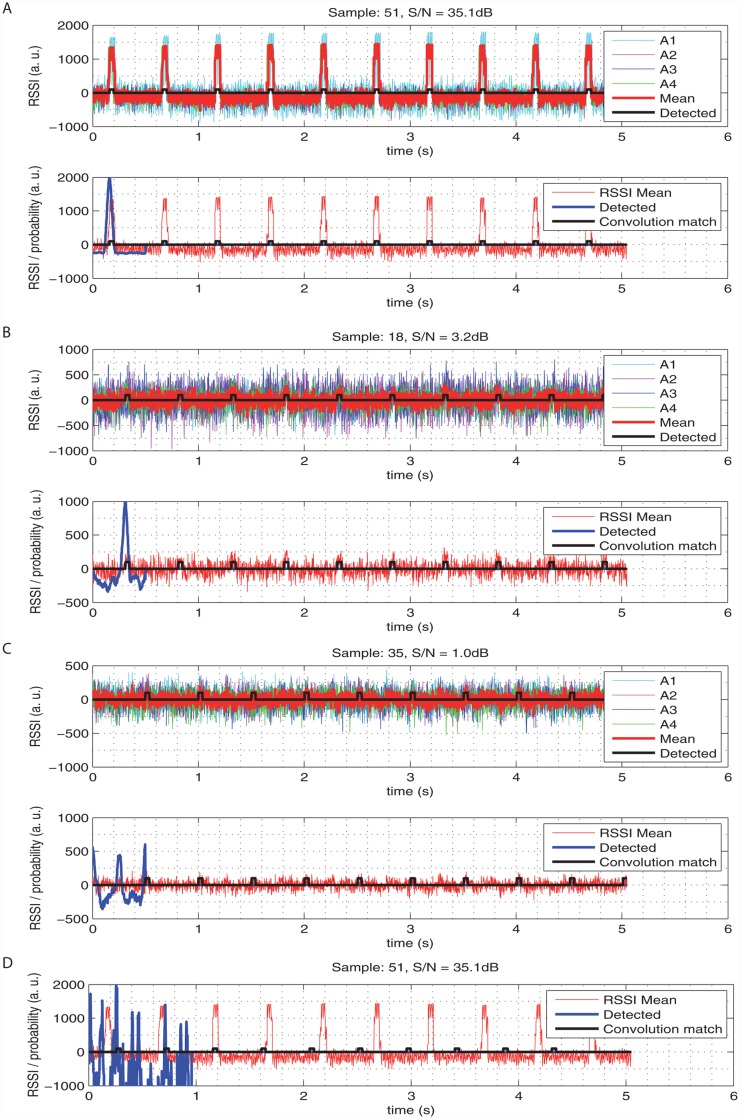
RSSI recording from individual antennas, averaged signal used for pulse detection (in red), the result of the convolution function (blue) and the best match (black): a) strong signal, good signal-to-noise ratio (35.1dB); b) weak signal, but recognisable (3.2dB); c) too weak signal (1dB), signal is lost and the detection fails; d) good signal but due to the inaccurate pulse timing, the detection fails.

The first step of the position evaluation is a detection of the repetitive pulse pattern as it is generated by the telemetry transmitter. For this step, the signals from all antennas are summed together to form an averaged RSSI recording (note the red waveform in Fig. 3a–d). Note that the averaging reduces the random noise in the RSSI and yields more data for further steps. Using a numeric convolution [23], this sum is then compared with the expected pulse waveform (given by known pulse timing), to detect the pulses in the noise background. The detected coincidence (the bold blue waveform in Fig. 3a–d) enables the estimation of the RSSI during the pulse (RSSI_on_) and during the silence period (RSSI_off_) and their difference is referred to as the signal-to-noise ratio. When the ratio, referred to as an acceptance threshold, is higher than ~4 dB (the precise threshold is estimated for each terrain deployment as shown later in this text), the position reading is considered valid. Fig. 3a shows the pulse detection on a strong signal; note that the result of the convolution function has one strong peak. Near the detection threshold (Fig. 3b), there is only a slight indication of the pulses in the averaged signal and almost no clue in the signals from individual antennas; the detection is still reliable. When the ratio is even lower (Fig. 3c), the pulses are indistinguishable from the noise. One very important aspect is the correct timing setting: the detection fails for signals with slightly changed pulse timing (by 5%, Fig. 3d) and indicates a low signal-to-noise ratio.

The next step is the azimuth calculation, which is calculated from mutual ratios of the RSSI on individual antennas as:

$$BRG={tan}^{-1}(\frac{RSSI_{L}+RSSI_{R}}{RSSI_{F}+RSSI_{Re}})+K$$

where the RSSI_on_ values are denoted relative to the antenna with the strongest RSSI reading: RSSI_F_ for front, RSSI_Re_ for rear antenna and left and right antennas relative to the front one. The *K* parameters denote the rotation of the front antenna from the north, i.e. *K* ∈ {0,90,180,270} degrees.

Since it is difficult to achieve an optimal spectrum in the rear quadrant of a Yagi-based directional antenna, the RSSI value of the weakest antenna RSSI_Re_, i.e., the one that actually reads the transmitter from the opposite side, is calculated from the other three values to avoid this issue:

$$RSSI_{Re}=-RSSI_{F}+\frac{RSSI_{L}+RSSI_{R}}{2}$$

At this point, the compensation of an imperfect antenna radiation pattern is applied. For each antenna array, the deviations in the azimuth evaluation caused are evaluated (see Fig. 2b) and are subtracted from the resulting BRG.

From the RSSI_F_, i.e., the strongest value, and the calculated bearing, the final RSSI is estimated, i.e., the estimated RSSI that would be read in case an antenna is directed towards the transmitter:

$$RSSI_{max}=\frac{RSSI_{F}-\frac{RSSI_{L}+RSSI_{R}}{2}}{cos(\Delta BRG)}+\frac{RSSI_{L}+RSSI_{R}}{2}$$

where ΔBRG denotes the deviation of the resulting BRG from the front antenna. Note that the RSSI is expressed as a logarithmic scale, in decibels: we use unit dBa, a power ratio that is referenced to one attowatt (aW), which is shifted by 150 dB to standard dBm. This means that a strong signal has ca 100 dBa and a weak one, ca 30 dBa. The power of received signal is given by the equation:

$$P_{rx}=cP_{tx}\frac{1}{d^{2}f^{2}}$$

The distance is calculated from the RSSI from the expected exponential attenuation of the electromagnetic signal in the terrain by the relationship:

$$DIST=AB^{RSSI_{max}}$$

where the coefficients *A* and *B* are usually calibrated for a particular deployment using beacon transmitters. Note that *A* defines the linear scaling and represents mutual ratio of distance and RSSI scale and B defines the exponential slope. Both parameters reflect the factory settings of individual stations as well as the external influences (e.g. terrain, height of placement).

### Detection of multiple transmitters (multiplexing)

To handle multiple transmitters, the BAARA system utilises frequency multiplexing, i.e., each transmitter is tuned to a unique frequency band. Individual frequency bands are scanned consecutively in a round-robin manner. Besides the frequency bands, the tracking station also provides the possibility to configure different pulse timings for individual channels. In the field, this is utilised to put transmitters with different timings on adjacent channels, to increase the robustness against cross-talk. The frequency division multiplexing is an advantage in case there are more transmitters within the range of stations; unlike, for example, digitally coded tags, the possible overlaps are not an issue. The location results are written to internal memory, together with the original RSSI values, so that the location can be precisely adjusted within the data post-processing.

### Portability and deployment

When designing the BAARA system, we also focused on the compactness and portability of the system. The total weight of the complete station is 2,915 g (including the battery and a solar panel).

The battery allows for three days of operation between replacements. When the accumulator is complemented by a solar panel in areas with minimal cloud (e.g., Egypt), there is virtually unlimited operating time. Note that the lead battery packs of the automated station can be supplied from any DC source of 9 to 24 V. Two persons need ~20 min to set up one station.

An important aspect is proper configuration of individual tracking stations as well as the transmitters. Besides the correct configuration of frequency channels, all parts need to have the system time closely matched to each other. Typically a NTP-synchronized (Network Time Protocol) PC is selected as a reference that is used to configure system clocks of all components.

### Transmitter

Traditional transmitters based on analogue circuitry are very sensitive to changes in ambient temperature and supply voltage as the battery is being discharged. These influences result in significant timing and frequency drifts that make fine-tuning of the receiving subsystem complicated.

To address this issue, we built a custom microcontroller-driven digital transmitter (Biotag, Institute of Scientific Instruments in Brno, ASCR), which is more robust against these drifts, mainly due to a precisely controlled timing. The microprocessor control also made it possible to incorporate a communication interface that allowed for configurable transmission parameters of each particular transmitter. The pulse period and its length are accurately maintained throughout the transmitter lifetime, which is important for automatic processing of signals. The newly built transmitter has two main advantages: i) the receiver can be narrowband and does not need to be refined during the transmitter operational period; and ii) a known precise timing is used to detect the correct signal. It is possible to adjust the transmitter frequency channel (over the entire frequency band), the period and duration of the pulses and the output power of the transmitter, to define several independent time-windows at different times of the day. This allows for a duty cycling, i.e the transmission can be switched off at a time when no information is required (e.g., during the day for bats) and thus, prolong the battery life.

The basic parameters of Biotag, as used for testing and calibration as well as for the field studies were following: the weight of hardware itself, including one 200mAh CR2030 battery and whip antenna is 7 g, configurable frequency range of each transmitter is 1,5MHz, observed lifetime with 15 hrs/day active cycle were 25 days.

### Data post-processing

After the positional data from the stations are collected and stored, the records from the BAARA tracking station undergo several processing stages: the positions are recalculated with a better precision than could be achieved with the station hardware and with an optional additional filtering. For each tracking station, the S/N ratio threshold is estimated on the basis of calibration measurements using a beacon transmitter with a known position, to reach an optimal false-accept/false-reject ratio.

In the next step, the records are used to calculate triangulations. In case there are more than two simultaneous records within the time window used for triangulation (typically 15 s to 5 min), the final position is detected as the centre of gravity of the minimum convex hull of all mutual intersections [24]. All the processing information is incorporated into a dedicated information system, BAARA IS (Prokeš et al., in preparation). New data are imported via GIS software (ArcGis 9.2, ESRI, USA) and further analysed with aid of Hawths Tools [25].

### Test and calibration measurement

We have conducted several experimental measurements to assess the localisation accuracy of the tracking stations, as well as to configure operational parameters. The aims were to determine the correct acceptance threshold, to inspect the accuracy of azimuth reading both in the laboratory and in the field and finally, to evaluate the distance estimation.

Before the field test was commenced a series of calibration measurement had been done in the laboratory. This comprised the verification of individual antenna’s radiation pattern and generation of customized correction vector for each antenna array, i.e. for each tracking station. In the next step, we inspected the behaviour of the convolution based detection of the pulses read from the telemetry transmitters.

The first measurement, aimed at the azimuth determination, was conducted on an outdoor testing polygon. The station was put onto a rotating flagpole so that the antenna array was approximately 5 m high. A custom-built signal generator (based on the same circuitry as the Biotag transmitter with added controls and attenuation stages), was placed on another flagpole at a height of four m and 40 m from the station. During the measurement, the station was gradually rotated by 15 degrees in the range of <0, 90> degrees and the azimuth reading and the RSSI parameters were tested.

The second measurement was carried out during the field deployment of BAARA in southern Turkey, near Adana. The station was placed on a hill peak with a clear view of the surroundings. One Biotag transmitter was fastened onto a four-m high flagpole that was mounted on a car. The car cruised in the station surroundings at a distance of 2–15 km during a 5-h period, while the station was configured to read the signals. The data were then used to demonstrate the method for acceptance threshold determination (for the specific deployment location) as well as azimuth reading testing.

The third measurement, aimed at inspecting the RSSI-to-distance relationship, utilised the same set-up as the first measurement, but the station was not rotated and the transmitter was gradually placed at a different height to show the impact on the measured RSSI.

Using the fourth measurement, we also compared the effective range of two transmitters; the controller-driven Biotag (effective radiated power 0.1 mW, 7 g of tested sample) and an analogue transmitter made by Holohil Systems Ltd. (model LB-2X, Ontario, Canada, effective radiated power 300 nW, 0.32 g of tested sample). The station was placed in a fixed position and the transmitters were placed at selected locations with an unobstructed view of the station at a defined distance.

Finally, the system has been deployed during the field trips, where it stand as an important support for studies of *R. aegyptiacus* model group.

### Material

During the laboratory testing of the azimuth reading, we obtained a total of 2,093 position measurements. The second (field) test yielded 1,550 position fixes, 971 of which were used for the azimuth reading accuracy assessment. Within the third test, we collected 150 records over 10 height steps. When we compared the efficiency range between different transmitters, we obtained 242 measurements for the Biotag and 181 for Holohil transmitter. During the telemetry sessions in Egypt, we tagged 111 fruit bats and received 55,691 locations during three field trips. Currently, the data have not been available through a public resource.

## Results

### Antenna array calibration

Antenna array of each station has been calibrated to mitigate the influence of mechanical and RF tuning imperfections. The calibration measurement incorporates the measurement of individual antennas’ radiation pattern (as shown in Fig. 2a) and the evaluation of azimuth calculation error (Fig. 2b). Then an inverse function of the calculation error is applied on all further calculations to cancel out the errors.

### Pulse detection verification

We have tested the validity of the pulse detection method. Sample signal waveforms (shown in Fig. 3) indicated, that the pulse detection is reliable once the useful signal is stronger than the noise background, independently on the absolute values. The inspection also reveals, that the detection method is also robust against signals with different timing (Fig. 3d), even a strong signal with 10% difference is filtered out (but it is added to the noise background). Generally, the necessary ratio of the useful signal to the noise background (referred to as acceptance threshold) was approximately 3dB ratio, the precise value was a subject of in-field testing.

### Azimuth error depends on the signal strength

**Fig 4:**
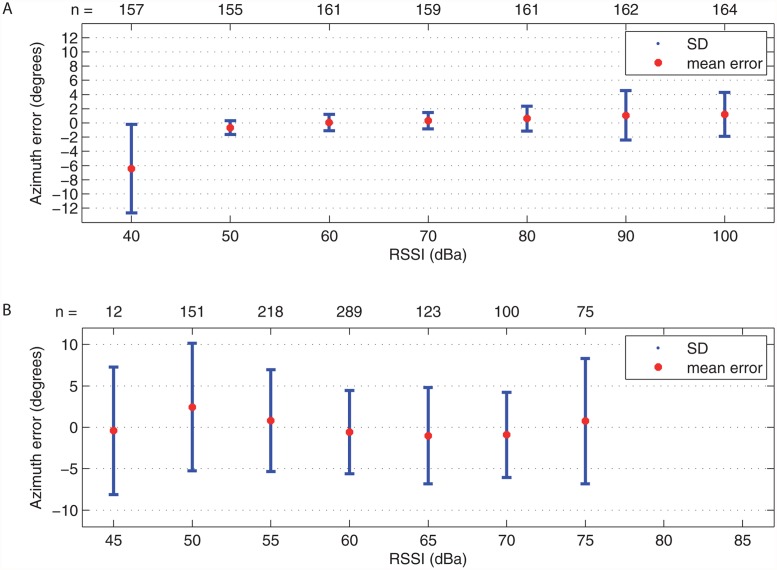
Azimuth reading error for different signal strengths (RSSI) a) in laboratory conditions and b) in the field. It is apparent that the azimuth calculation error is significantly higher in the field due to the environmental aspects.

The results from measurement under nearly ideal conditions (referred to previously as the first measurement), featured a relatively stable performance when the RSSI (Received Signal Strength Indication) level lay between 50 and 100 dBa, where the mean azimuth error was less than one degree (Fig. 4a). The standard deviation increased from less than ± 2 degrees for medium-level signals (50–70 dBa) to less than ± 5 degrees for strong signals (>80 dBa). The weak signals exhibited an accuracy of 6.2 ± 6.3 degrees.

The worst results for strong signals were shown by the non-linear transfer characteristics of the receiver RSSI detector. The weak signals were probably due to the increased influence of noise and possibly to an unwanted interference.

The results from the in-field testing (referred to as the second part of the second measurement), showed the azimuth determination error, including the influence of local terrain conditions (Fig. 4b). The result was 0.2 ± 6.2 degrees, whereas the mean error varied slightly in the range ± 2.3 degrees, which was probably due to the specific terrain shape.

Most field locations (71.5%) were acquired within the stable performance region between 45–100 dBa (Table 1). Nearly all locations <45 dBa were on feeding trees verified by the homing-in method (82% of them). Measurements with more than 100 dBa were excluded from the dataset during post-processing.

**Table 1:** Signal strengths (RSSI) values in the material from Egypt.

*RSSI (dBa)*	*number of fixes*
< 45	13,742
45–50	8,710
51–60	3,133
61–70	6,357
71–80	5,691
81–90	4,370
91–100	11,584
>100	2,104
**total**	**55, 691**

### The acceptance threshold assessment

**Fig 5:**
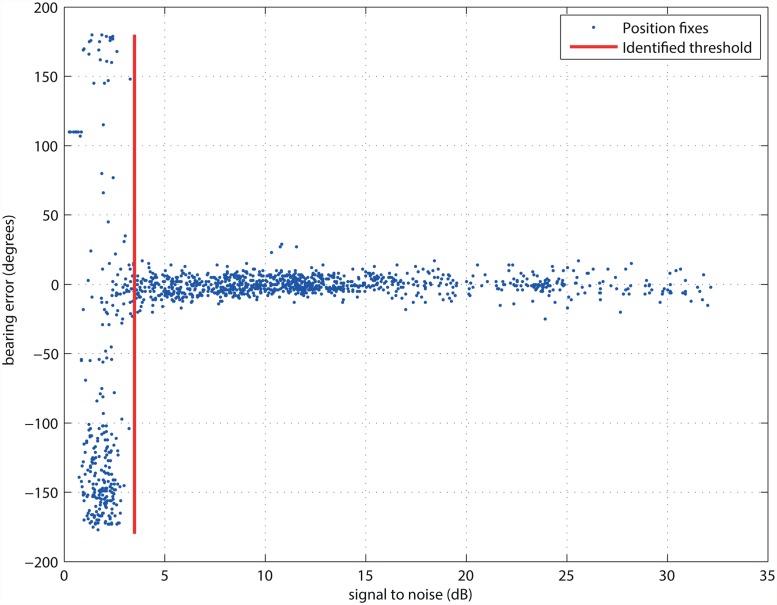
Acceptance threshold identification on the basis of field-acquired data. The threshold reflects the fact that once the signal to noise ratio becomes too low, the pulse identification fails and the rate of errorneously identified positions rises sharply.

As a part of the second experimental measurement, we measured the relationship between the indicated signal-to-noise ratio and azimuth estimation error (Fig. 5). The plot clearly shows a dramatic increase in the azimuth error for values below 3dB, therefore, the value 3.3dB was considered a safe value for the threshold.

The measurement was conducted for each station at specific deployment locations, however, experience has shown that the results were very similar: for two telemetry sessions with five deployed stations, the acceptance threshold fell to within the range of 3.0–3.5 dB.

### Distance estimation

**Fig 6:**
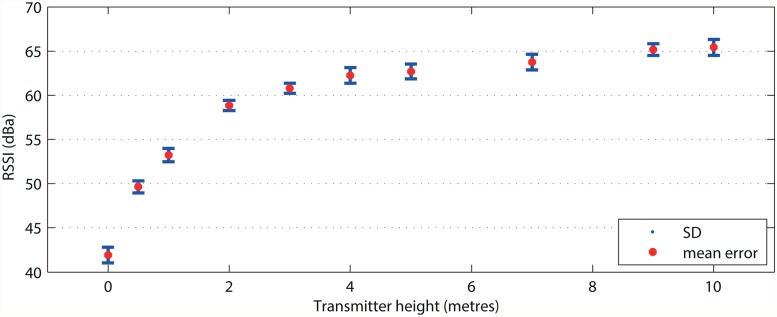
Different signal strengths (RSSI) at different heights of the Biotag above ground. The results indicate that first 3 meters of altitude upon the ground significantly attenuates the signal due to obstruction of the Fresnel zones.

The third measurement showed that the signal strength increased rapidly when we installed the transmitter at a height of 4 m above the ground. However, the RSSI values remained constant at heights from 4 to 10 m (Fig. 6).

**Fig 7:**
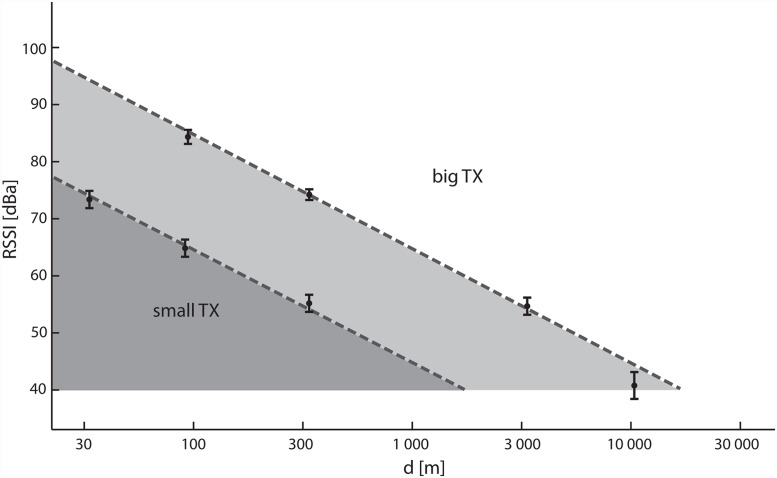
Modelling of the signal propagation of two different transmitters (small TX, Holohil; big TX, Biotag) in the field. The solid line represents the theoretical value of the effective range in free space. The values in the box-plots represent individual measurements under ideal conditions (line of sight). Other measured values fall into the grey area below the line. For the weaker transmitter (Holohil), the grey zone is shifted towards a smaller range.

Signal propagation, as measured in the fourth measurement, was described by equation and visualised by a straight line (Fig. 7). The line represents the theoretical value of the free-space propagation [14], and at the same time, presents the strongest possible signal, when the values measured in the field fill in the space below this line.

### Field deployment

**Fig 8:**
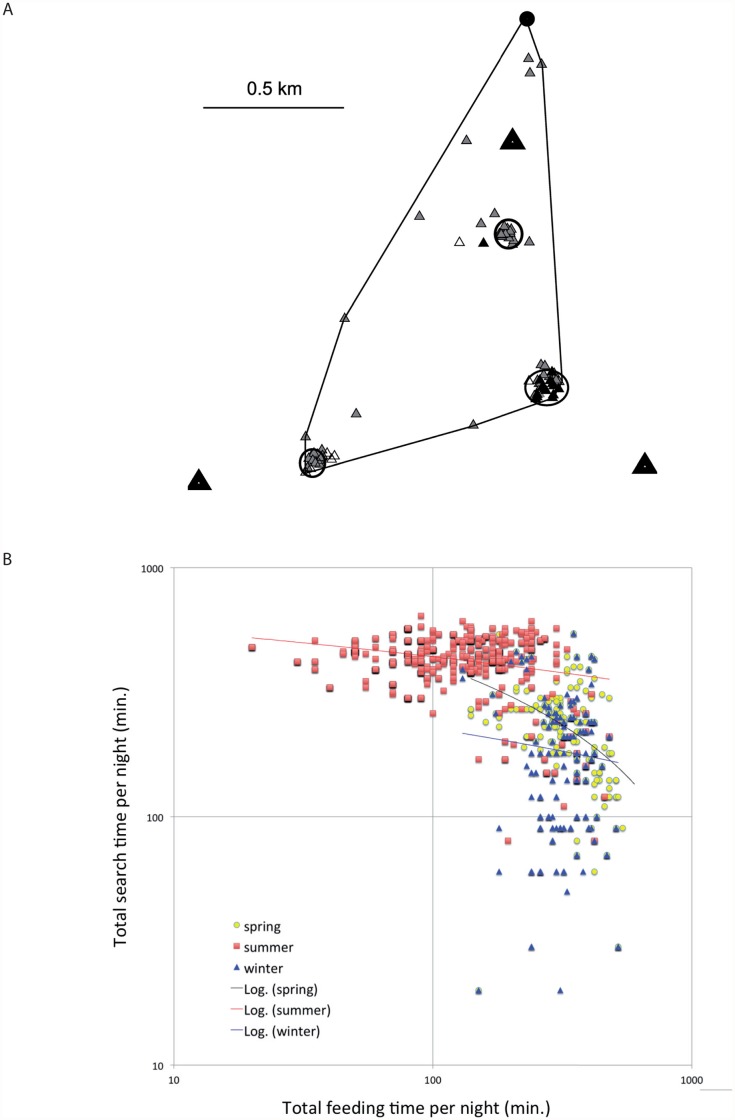
Spatial activity pattern of fruit bats. a) Triangulated locations of the representative fruit bat, *R. aegyptiacus*, tracked for one night during field tests of the BAARA automated radiotracking system. Black dot—roost, large black triangles—automated stations, small triangles—locations (n = 384), solid line—100% minimum convex polygon, elipses—kernel estimations 50% contour lines. Different colours show the level of triangulation: white—no triangulation; grey—by two; and black—by three stations. There are three feeding sites, isolated locations mark the commuting bat. b) Spatial activity pattern of a fruit bat population in Dakhla oasis, Egypt, in different periods of the year as revealed by instrumental record of automated radiotracking. *The feeding time* is a period with no changes in successive positioning of an individual in a record of single BAARA station, *the search time* is a period of changing successive positioning. Each sign expresses the sum of the respective two variables in a whole night record of an individual from a single BAARA station.

Finally, the system was deployed in the Dakhla oasis in Egypt during three field trips (winter 2010, spring and summer 2011), where we consecutively tagged *R. aegyptiacus* in a study area covering 10 × 10 km. Five stations took bearings every 2–10 min, based on the number of simultaneously followed bats. When more stations took a bearing of a particular transmitter, its precise location was calculated by triangulation during the post-processing of data (Fig. 8).

## Discussion

This section is organized as follows. A brief summary of wave (radio signal) propagation principles lays a foundation for discussing the azimuth and distance determination accuracy. Then the specifics of automated and manual tracking are discussed as well as compatibility issues and further design considerations.

### Characteristics of wave propagation

The Fresnel zone ellipsoid defines how much clearance is required for wave propagation. Some obstruction of the Fresnel zones can often be tolerated and the maximum obstruction allowable is 40% [14]. An obstruction of the first Fresnel zone has two consequences: the obstacles reduce the power of the received signal and any reflections cause the signal to scatter and propagate in multiple paths, so that waves can arrive from a different direction than the axis of the line of sight [15]. For instance, for the frequency band of 150 MHz (Biotags) and a distance of 2 km, the Fresnel zone ellipsoid will have a diameter of 63 m at the centre of the line of sight [16].

The location of a tagged animal, determined either manually or by the automated system, is represented by a combination of the two variables; direction and distance, relative to the point of observation.

### Accuracy of the direction determination

The direction is estimated from the mutual RSSI ratio from the antennas, supposing that a maximum strength is observed for a line of sight. The bearing accuracy is not principally affected by the signal attenuation caused by terrain, but when significant part of the signal arrives from a different direction than a line of sight. The multipath propagation is caused by a reflection of the signal by a substantially large mass of conductive material (e.g., wet hill, large metal fence). Simultaneous reception of the signal from multiple paths might cause interference, so that a minor change in the transmitter position might cause significant changes in the RSSI [14, 15]. Considering that the animals from the model group reside near the surface, even a very flat terrain might obstruct the signal path and cause the attenuation and multipath propagation of the radiosignal.

### Accuracy of the distance estimated from RSSI

The distance is estimated from the maximum RSSI, supposing that the signal is attenuated based on the length of the radiosignal path. These assumptions hold under optimal conditions when there are: (i) no obstacles in the space between the transmitter and receiver (typically defined by the first Fresnel zone); and (ii) no reflective surface (for particular frequency bands) near that space [14,17].

Attenuation is a consequence mainly of the absorption of the RF signal energy by obstacles (e.g., trees, large buildings) and the fading caused by the multipath propagation. From the point of view of the receiving side, the transmitter then appears to be more distant than it really is. Moreover, the RSSI then varies with the varying height of the transmitter and receiver, or more precisely the antenna, above ground [17–19].

The effective height of the transmitting and receiving antennas for the signal with 150 MHz is 20 m. This antenna height reduces attenuation at a distance of 2 km [19]. If the height of the transmitter and/or receiver antennas is less than 20 m, the wave energy is strongly influenced by the terrain. We found that the influence of the terrain is greatest if the antenna of the station is situated below 5 m above the ground. Subsequently, the signal attenuation is significantly higher than it would otherwise be, compared to broadcasts in free space. During tests of the automated stations, fruit bats flew near the feeding habitats at a minimum height of 8–10 m above the ground. While commuting between feeding habitats and roosts, fruit bats used high flight altitudes (mean 45 ± 27 m according to our observation with GPS (Global Positioning System) tags or 84.0 ± 27.4 m in [20]). Stations were installed on poles 4 m above the surface. In Egypt, antennas were placed at 12.2 ± 2.9 m, slightly lower than the effective height, because there was a very flat landscape. However, field tests showed that values of RSSI were relatively stable and suitable between 6 to 10 m.

### Achieving the best accuracy

The aforementioned paragraphs imply that the character of the study area where the monitoring system is deployed has a major impact on the system accuracy. Since the exact statement of the influence is impossible to determine, the only feasible approaches are prevention of the negative influences (where applicable) and consistent calibration of the system components for particular conditions.

The a-priory mitigation of negative influences comprises carefully selected spots for the tracking stations where the line of sight is preserved, i.e. the terrain is free of obstacles, large sources of RF noise, reflecting surfaces etc.

To a certain extent, the position estimation could be refined by calibration for particular terrains and deployment schemes. Radio beacons with known location (either stationary or mobile), simulating a transmitter mounted on a studied individual, might be deployed on known positions (over the study area) so that at least a coarse knowledge of the RF signal behaviour in the area could be determined and then used to compensate the position read by the tracking stations. Additional precision is achieved by an aggregation of positional data from multiple sources, e.g., the triangulation between time-coinciding positional fixes from more stations or comparison with manually obtained homing positions. Although many factors make the position estimation ambiguous, the automatic tracking approach allow for the collection of a significantly larger number of positions, so that an error of a single position is corrected by a massive repetition of the measurement, i.e. the error is compensated by a statistical means.

### Lack of manual and automated tracking

Automated measurements appear to be a suitable alternative that reduces the deployment of human operators. Complete replacement of the human factor is not appropriate or even possible in many cases. If the positioning of the transmitter (mounted on an animal) is a principal aim of the research, then the introduction of automation is feasible. The BAARA system replaces the human operators that determine the position of the transmitter at a distance and then specify it using triangulation. However, the system is not able to fully replace the observer using the “homing-in” method, whose goal is to obtain the minimum distance from the tracked animal. In such cases, a person is able to simultaneously detect a wide range of additional information, which adds another dimension to the research, e.g., how animals search for places where they stop for some time and how they drink or eat, what they eat, whether feeding places visited by other unmarked individuals and whether the local food supply is large or limited. These biological data essentially complete the understanding of the strategies of the studied animals.

The limit of standard tracking has always been the sample size, i.e., the number of simultaneously tracked individuals. The BAARA system allows automatic tracking of a theoretically unlimited number of individuals. However, for mobile groups (such as bats), it is practical to monitor 50 or more different channels/frequencies simultaneously. Thus, it is possible to obtain large datasets without pseudoreplications caused by the distribution of tracking research in multiple seasons, which cannot rule out re-tagging of the same animals relating to the structure of research over a longer period.

### Compatibility with third-party transmitters

A parameter-evaluation system was necessary to change the station every day with regard to unstable, changing parameters, such as the battery-strength and ambient temperature, and especially the time between pulses (LB-2N, Holohil System Ltd., Ontario, Canada). The software in the station is set to consider the signal as correct when it is within a period of a certain range of pulse (we use a ±3% tolerance). This range can be altered (increased), but this means a reduction of system resistance against interference. The receiving frequency can also be automatically tuned, e.g., every few hours, to identify the frequency with the strongest signal. However, scanning a large range of frequencies is associated with a time delay. Scanning must be repeated several times and the values averaged, excluding extremes resulting from placing the transmitter in places where wave propagation is not standard.

A better option would be miniaturisation of digital micro-controller-driven transmitters (e.g., the Biotag). Regarding the size and power consumption processor, miniaturisation (less than 0.5 g) is still a challenge. However, we have also developed improved algorithms for the estimation of the direction, which offer substantial improvement, to give a mean deviation of below 10 degrees. The novel algorithms also optionally allow for variable timing characteristics of the radiosignal, thus, it should perform better in combination with third-party transmitters.

### New objectives

The BAARA system was designed for robust operation under various field conditions and to optimise the maximum weight and storability for easy transport. For this reason, a number of planned features have not yet been implemented in the presented prototype. The processing unit currently consists of several sub-modules and the system is ready for the addition of new modules to extend the functionality. One of these modules could be a GSM/GPRS (Global System for Mobile communication/General Packet Radio Service) bridge module (any off-the-shelf available product, e.g., the RouterBoard by Mikrotik, www.mikrotik.lv) for remote access, where a wireless protocol for configuration and data interface could be implemented. Such a module would allow, for example, a real-time overview of the monitoring of animal movements and remote maintenance, without regular visits to each station and manual data downloading.

We also consider that alternative antenna modules—e.g., more antennas in a circle with a better radiation beam—might improve the operating range in specific situations. A multi-element directional Yagi antenna located only in two directions, e.g., to monitor a commuting corridor might also be local. The logical direction of future development will be the further miniaturisation of stations and an increase in their number, leading to a coverage of larger areas and thus to a more efficient triangulation.

Another area of development are the transmitters and system interoperability with third-party transmitters. We intend to adjust the system to operate with a wider range of telemetry transmitters, among others with coded ones. The pulse coding is also a possible extension for Biotags together with further enhancement of the lifetime using better power control and/or advanced energy sources, such as a new batteries (e.g. [21])

### A novel perspective in the study of spatial behaviour: the population approach

The information provided by traditional techniques of radiotracking is for obvious reasons restricted to the domain of the spatial behaviour of an individual [22, 23]. Here, we illustrate this with a simple comparison of seasonal differences in the activity pattern of the fruit bat population in Dakhla oasis (Fig. 4b), inferred directly from the primary instrumental data. Without discussing details and biological meanings of the record (which will be surveyed elsewhere), we stress that even rough instrumental data provide impressive information on seasonal differences in population activity patterns and patterns of individual variation. Supplementing such data with a series of contextual variables allows the potential to rigorously test the effects of various intrinsic factors that modify the spatial behaviour of a mobile target species and/or responses of a population to variations in diverse environmental cues. In other words, the system of automated radiotracking provides a novel type of data, which promise to enlarge the outcome of radiotracking studies and can essentially contribute to analyses of adaptive dynamics of a population and other phenomena in the domain of population ecology.

## Supporting Information

S1 Data Azimuth calculation error (denoted as BRG error) of four antenna arrays (data 1–4). (ZIP)

S2 Data Values of RSSI recording from individual antennas, a) strong signal, good signal-to-noise ratio (sample 51); b) weak signal, but recognisable (sample 18); c) too weak signal (sample 35) and d) good signal but due to the inaccurate pulse timing, the detection fails (sample 51). (ZIP)

S3 Data Azimuth reading error for different signal strengths (RSSI) in laboratory conditions and in the field (Fig. 4) and acceptance threshold identification (Fig. 5). (XLS)

S4 Data Values of signal strengths (RSSI) at different heights of the Biotag above ground. (M)
